# Maternal educational inequalities in child’s birth weight: ONS Longitudinal Study

**DOI:** 10.1093/eurpub/ckac131.120

**Published:** 2022-10-25

**Authors:** J Pikhartova, N Shelton

**Affiliations:** 1 Epidemiology and Public Health, University College London, London, UK; 2 RECETOX, Masaryk University, Brno, Czechia

## Abstract

**Background:**

Studies found that maternal education is the most frequently assessed predictor of child’s birth weight. Lower level of education was consistently found as indicator of lower child’s birthweight. In turn, inequalities in child’s birthweight have been repeatedly shown to be closely related to inequalities in later adult health. The aim of this project is to evaluate the association between the highest achieved level of maternal education and birthweight of single born babies in large English and Welsh population sample, taking into account factors such as child’s gender, parity, maternal age, partnership status, ethnicity, and household socioeconomic characteristics.

**Methods:**

Using Office for National Statistics Longitudinal Study (ONS LS) based on English and Welsh Census data, information from almost quarter of a million children born since 1981 to ONS LS sample mothers were used. Maternal education was categorised in 3 categories (below secondary, completed secondary education, degree and higher), and its association with child’s birth weight was analysed by logistic regression accounting for range of available covariates.

**Results:**

Significant association between the level of education and birth weight was found in crude analysis (p < 0.001). When adjusted, the magnitude of the association with education gradient declined but remained highly significant and was found to considerably increase over the years. The birth weight difference between those born to mothers with below secondary education and those born to mothers with degree increased by more than 60 grams (p for change <0.001) between 1981 and 2016.

**Conclusions:**

These findings support previous evidence based on different population samples. According to our results, children of mothers with below secondary education tend to have lower birth weight. Our results suggest that the inequalities in birth weight by the highest education level achieved by mother significantly increased since 1981.

**Key messages:**

• Lower levels of maternal education predict low birth weight in children.

• The differences in birth weight by maternal educational demonstrate increase in inequalities over the years.

